# Next-generation sequencing in childhood-onset epilepsies: Diagnostic yield and impact on neuronal ceroid lipofuscinosis type 2 (CLN2) disease diagnosis

**DOI:** 10.1371/journal.pone.0255933

**Published:** 2021-09-01

**Authors:** Kimberly Gall, Emanuela Izzo, Eija H. Seppälä, Kirsi Alakurtti, Lotta Koskinen, Inka Saarinen, Akashdeep Singh, Samuel Myllykangas, Juha Koskenvuo, Tero-Pekka Alastalo

**Affiliations:** 1 Blueprint Genetics Inc, Seattle, Washington, United States of America; 2 Biomarin Pharmaceutical, Novato, California, United States of America; 3 Blueprint Genetics Oy, Espoo, Finland; The Islamia University of Bahawalpur, PAKISTAN

## Abstract

Epilepsy is one of the most common childhood-onset neurological conditions with a genetic etiology. Genetic diagnosis provides potential for etiologically-based management and treatment. Existing research has focused on early-onset (<24 months) epilepsies; data regarding later-onset epilepsies is limited. The goal of this study was to determine the diagnostic yield of a clinically available epilepsy panel in a selected pediatric epilepsy cohort with epilepsy onset between 24–60 months of life and evaluate whether this approach decreases the age of diagnosis of neuronal ceroid lipofuscinosis type 2 (CLN2). Next-generation sequencing (NGS)-based epilepsy panels, including genes associated with epileptic encephalopathies and inborn errors of metabolism (IEMs) that present with epilepsy, were used. Copy-number variant (CNV) detection from NGS data was included. Variant interpretation was performed per American College of Medical Genetics and Genomics (ACMG) guidelines. Results are reported from 211 consecutive patients with the following inclusion criteria: 24–60 months of age at the time of enrollment, first unprovoked seizure at/after 24 months, and at least one additional finding such as EEG/MRI abnormalities, speech delay, or motor symptoms. Median age was 42 months at testing and 30 months at first seizure onset; the mean delay from first seizure to comprehensive genetic testing was 10.3 months. A genetic diagnosis was established in 43 patients (20.4%). CNVs were reported in 25.6% diagnosed patients; 27.3% of CNVs identified were intragenic. Within the diagnosed cohort, 11 (25.6%) patients were diagnosed with an IEM. The predominant molecular diagnosis was CLN2 (14% of diagnosed patients). For these patients, diagnosis was achieved 12–24 months earlier than reported by natural history of the disease. This study supports comprehensive genetic testing for patients whose first seizure occurs ≥ 24 months of age. It also supports early application of testing in this age group, as the identified diagnoses can have significant impact on patient management and outcome.

## Introduction

Neurologic and metabolic disorders that include epileptic seizures are among the most common genetic disorders presenting in early childhood. Major improvements in sequencing technologies during the last two decades have significantly improved our understanding of the molecular genetic origins of epilepsies and sparked a new era in the genetic diagnosis of epilepsy. The etiology of epileptic seizures can be difficult to discern. Although epilepsy can result from trauma, tumors or infections, a significant number of patients with epilepsy are thought to have a causative genetic factor [1,2].

The spectrum of epilepsies can vary from self-limited and treatable to severe, progressive, drug-resistant forms. In addition to channelopathies and neurotransmitter receptor and transporter defects as causes of epilepsy, >300 inborn errors of metabolism (IEM) can present with epileptic seizures, adding a further layer to the differential diagnosis [3]. Given the complexity of the disease spectrum, the limitations of traditional genetic diagnostics, the lack of recently updated recommendations on genetic testing in pediatric epilepsy, and limited access to comprehensive genetic testing, it is not surprising that patients often experience years of delay before a genetic diagnosis is established and the underlying etiology is understood. Nonetheless, early molecular diagnosis is essential for the personalized management and targeted therapy of epilepsy and improved outcomes [4].

Several studies have addressed the utility of next-generation sequencing (NGS) in the diagnosis of epilepsy. These studies varied in terms of the patient cohort size, patient demographics and phenotypic spectrum, the testing strategy, the variant interpretation approach, and the quality and performance of the NGS technology. These factors contribute to the variability observed in the diagnostic yields and conclusions. The majority of these studies have focused on patients with early-onset seizures (birth to two years) or have analyzed unselected cohorts of patients with large variability in age of onset, severity, and outcome. Studies with cohorts of >100 patients [5–11] have generally demonstrated a lower diagnostic yield when compared to smaller studies [12–17]. Several studies have provided evidence that the molecular diagnostic yield is strongly correlated to the age of onset and epileptic syndrome, with the highest yield being among patients with developmental and epileptic encephalopathies (DEE) and seizure onset in the neonatal period.

As many NGS studies have focused on early-onset and severely affected patients, our understanding about the genetics of patients with seizure onset after two years of age is limited. Only a few studies have specifically addressed this age group, and those had small patient numbers. Oates et al reported a 4% diagnostic yield in 46 patients with seizure onset after two years of age and suggested that NGS panels are not cost-effective in this patient group [18]. In another study, Moller et al demonstrated a 14% diagnostic yield in a cohort of 29 patients with seizure onset between two and nine years [19]. Both studies demonstrated lower diagnostic yields when seizure onset was after two years of age. In summary, studies addressing the clinical utility of genetic diagnostics for patients with seizure onset after the neonatal period, and especially after two years of age, are lacking.

The goal of this study was to evaluate the clinical utility and diagnostic yield of NGS-based genetic testing in a patient age group that has not been studied extensively in the past. Patients with ages ranging from 24 to 60 months of life with their first unprovoked epileptic seizure between 24 and 60 months of age were eligible for the program. We also evaluated whether this genetic diagnostic approach could decrease the age of diagnosis of neurodegenerative diseases such as neuronal ceroid lipofuscinosis (CLN2).

## Materials and methods

### Cohort

The cohort included 211 consecutive patients referred for genetic testing as part of the BioMarin Pharmaceuticals Inc. sponsored testing program in 2018 and 2019 (https://blueprintgenetics.com/beyondpaediatricepilepsy/). The testing program was offered in European and Middle Eastern countries (68 countries). Thirty-four patients (16.1%) were from Middle Eastern countries (Saudi Arabia, Oman, Kuwait and United Arab Emirates), 19 patients (9.0%) were from Israel, and 158 (74.9%) were from Europe. Patients had to be between 24 and 60 months of age and had to have their first unprovoked seizure between 24 and 60 months of age. Additionally, patients had to have at least one of the following findings: abnormal MRI (such as, but not limited to, cerebellar atrophy, cerebral atrophy, periventricular white matter hyperintensity) abnormal EEG, history of language delay or regression, motor impairment or regression. In addition, the requisition form completed by the referring physician also allowed them to specify additional findings in their patient such as developmental delay, behavioral abnormalities, sleep disturbances, visual impairment and other sensory impairment. These features were not defined as they were not required for inclusion in the study nor were they mandatory fields for completion of the order form. Detailed clinical information regarding the patients was not available to the authors.

Referring physicians were responsible for determining that their patient met eligibility criteria and were required to sign a statement to this effect. Testing did not proceed unless the patient met eligibility criteria and the signed statement was received. The study did not stipulate specific exclusion criteria, however, sites were encouraged to enroll patients with a suspected genetic etiology for seizures to ensure maximum benefit for those tested.

Informed parental consent was obtained by the ordering health care provider and attested to in writing for all patients who participated in this testing program. This work was reviewed by the Western Institutional Review Board (IRB) and received an excemption determination (WCG IRB Work Order #1-1379311-1).

### Next-generation sequencing

The study was initiated with a 194-gene panel (S1 Table); 25 patients (11.8%) were analyzed with this panel. The 194-gene panel analysis was done using the OS-Seq™ (oligonucleotide-selective sequencing) NGS method on the NextSeq sequencing system (Illumina) [20]. In this analysis, the mean sequencing depth was >170x and 99.6% of target nucleotides were covered with >20x sequencing depth. The remaining 186 patients (88.2%) were analyzed with a 283-gene epilepsy panel (S1 Table). The 283-gene panel was carved out of an in-house tailored IDT-based whole-exome sequencing platform performed on the NovaSeq sequencing system (Illumina). The mean sequencing depth was >200x and 99.6% of target nucleotides were covered with >20x sequencing depth. The platform included custom oligonucleotides targeting 70 non-coding/deep intronic variants associated with epilepsy (S2 Table).

Sequencing-derived raw image files were processed using a base-calling software (Illumina) and the sequence data was transformed into FASTQ format. Clean sequence reads of each sample were mapped to the human reference genome (GRCh37/hg19). Burrows-Wheeler Aligner (BWA-MEM) software was used for read alignment. Duplicate read marking, local realignment around indels, base quality score recalibration and variant calling were performed using GATK algorithms (Sentieon).

### Sanger sequencing

Bi-directional Sanger sequencing was used to confirm likely pathogenic (LP) and pathogenic (P) sequencing variants. All primers are available upon request. The sequence variant analysis pipeline was validated in the CLIA- and CAP-accredited Blueprint Genetics diagnostic laboratory.

### Copy-number variant analysis

Copy-number variant (CNV) analysis was performed bioinformatically concurrently for all patients from the NGS data using a commercially available bioinformatic pipeline CNVkit [21] and an in-house developed deletion caller based on read-depth to improve the detection of small CNVs. Further information about this proprietary deletion caller is available upon request. All heterozygous CNVs affecting < 10 target exons and hemizygous deletions < 3 target exons were confirmed using quantitative-PCR assays. The CNV analysis pipeline was validated in the CLIA- and CAP-accredited Blueprint Genetics diagnostic laboratory. The sensitivity to detect single exon deletions was validated to be 71.5% (NextSeq OS-Seq assay) and 92.3% (NovaSeq WES assay).

Variants were classified according to a point-based, modified adaptation of the Association for Molecular Pathology/American College of Molecular Genetics and Genomics guidelines [22] with evidence from population and gene-/disease-specific databases, in silico prediction tools (including PolyPhen [23], SIFT [24] and Mutaster [25]), our in-house variant database, multiple publicly and commercially available mutation databases and appropriate scientific literature as the foundation for scoring) as outlined in the Blueprint Genetics website (https://blueprintgenetics.com/variant-classification/). The Blueprint Genetics classification scheme is similar to the ACMG/AMP guidelines in that multiple independent lines of evidence must be met (e.g. rare in population databases, predicted deleterious by *in silico* software, segregation with disease, *de novo* in a patient with no family history, damaging impact shown in well-established functional studies, etc.) to achieve a likely pathogenic or pathogenic classification, and several of these criteria are equivalent in both schemes. A test result was considered diagnostic when the patient was found to have one or two pathogenic or likely pathogenic variants in a single gene, depending on the mode of inheritance. Highly suspicious variants of uncertain significance (VUS) are listed in Table 4. These VUS were considered suspicious based on 1) a strong and specific correlation between the gene and patient’s phenotype, 2) the variant being novel or extremely rare in the Genome Aggregation Database (gnomAD) control cohorts, and 3) *in silico* predictions supporting pathogenicity or the amino acid position in question being highly conserved in mammals and evolutionary more distant species, suggesting that the position does not tolerate variation. All reported variants were de-identified and shared in the ClinVar database.

## Results

A total of 211 patients were included in this study. The mean age was 3.5 years (42 months [range 24–60 months]) (Table 1). Males represented 58.8% of the group. The mean age at epilepsy onset was 2.5 years (30 months [(range 24–57 months]). The mean time from seizure onset to taking part in this genetic testing program was 10.3 months. At the time of inclusion, language delay was reported in 70.1% of patients, motor disturbance in 55.9%, and developmental delay in 48.3%. In addition, 42.7% of patients had abnormal EEG and 30.8% had abnormal MRI findings. Behavioral abnormalities were described in 41.7% of patients, sleep disturbances in 25.6%, and vision impairment in 10.4%. A family history of seizures was reported in 16.6% of cases.

**Table 1:** Patient demographics.

	Total cohort (n = 211)	Diagnosis (n = 43)	Suspicious VUS (n = 17)
Male/Female (%)	58.8/41.2	41.9/58.1	76.5/23.5
Mean age in months (range)	42 (24–60)	44 (24.5–59)	46 (25–60)
Mean age at seizure onset in months (range)	32 (24–57)	29 (24–57)	30 (24–52)
Mean time from seizure onset to genetic test in months (range)	10.3 (0–35)	10.0 (0–33)	12.9 (1–35)
Language delay	70.1%	79.1%	88.2%
Motor disturbance	55.9%	79.1%	64.7%
Developmental delay	48.3%	65.1%	52.9%
Abnormal EEG	42.7%	46.5%	35.3%
Abnormal MRI	30.8%	48.8%	35.3%
Behavior abnormalities	41.7%	44.2%	58.8%
Sleep disturbances	25.6%	25.6%	23.5%
Visual impairment	10.4%	11.6%	23.5%
Family history of seizures	16.6%	11.6%	29.4%

VUS, variant of uncertain significance; EEG, electroencephalography; MRI, Magnetic resonance imaging.

In this study, we established a diagnosis in 43 patients, resulting in a diagnostic yield of 20.4%.(Table 1). We detected a highly suspicious VUS in an additional 17 (8.1%) cases. These are variants that would be reclassified as likely pathogenic if they are shown to be *de novo* in a proband with autosomal dominant disease or if in trans with a known disease-causing variant in a proband with autosomal recessive disease. The demographic and clinical characteristics of all categories (total cohort, diagnosis, and suspicious VUS) are listed in Table 1.

**Fig 1:**
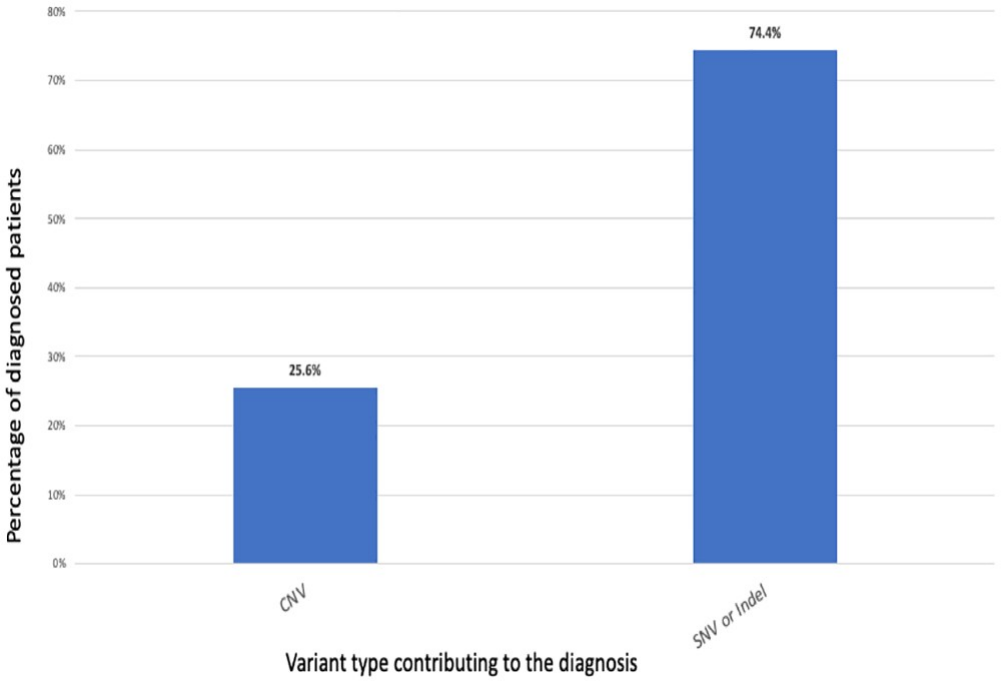
The contribution of CNVs, SNVs and Indels in patient’s receiving a diagnosis.

Among the 211 patients tested, our NGS technology detected a pathogenic or likely pathogenic CNV in 11 patients (5.2%) (Table 2). In the diagnosed cohort, 25.6% of patients were identified to have a disease-causing CNV (Fig 1). The deletions ranged in size from a 242bp intragenic deletion to a 9Mb deletion affecting a number of genes. The smallest deletion was a 242bp deletion in *CACNA1A*. It was identified in a child (ID 01) with myoclonic and atonic seizures, hemiparesis, ataxia, and delayed speech. A heterozygous single exon deletion of similar size was detected in *PPT1* together with a pathogenic nonsense variant p.(Arg151*) in a patient (ID 10) originally suspected to have Angelman syndrome who suffered from atonic seizures, motor disturbances and developmental delay. This was the only patient who was found to have both a disease-causing CNV and SNV. A 766bp hemizygous deletion in *SLC6A8*, affecting exons 10–13, was detected in a child (ID 02) with speech delay and focal seizures starting at the age 36 months. A 122,226bp deletion in *MEF2C*, affecting the full protein coding region of the gene, was found in a child (ID 09) with language delay, abnormal MRI and EEG findings, and seizure onset at 26 months. A heterozygous 2.1Mb deletion involving the short arm of chromosome 1 was detected in a child (ID 03) with treatment-resistant epilepsy with onset at 27 months, severe intellectual disability, global developmental delay, scoliosis, microcephaly, and dysmorphic features. This deletion partially overlaps the 1p36 deletion syndrome and includes 37 genes, including seven OMIM Morbid genes: *CEP104*, *GABRD*, *GNB1*, *PEX10*, *PRDM16*, *SKI*, and *SMN1*. Mowat-Wilson syndrome, caused by a 9Mbp deletion in chromosome two including all of *ZEB2*, was identified in a four-year-old girl (ID 07) with developmental delay, microcephaly, and seizure onset at 28 months. Three patients (IDs 04, 05, and 06) harbored heterozygous chromosome 15 deletions, ranging from 4.87 to 6.32Mbp, involving the Angelman/Prader-Willi syndrome critical region. All of these patients had developmental delay and seizure onset at 24 months of age. Interestingly, a patient (ID 08) with developmental delay and seizures was diagnosed with a 72Mbp duplication (1q25.2-q44) involving 786 genes on chromosome one and a 40Mbp deletion (Xq23-q28) involving 480 genes on the X chromosome, suspicious for an unbalanced translocation. Lastly, one patient (ID 11) was found to have a 772kb deletion on chromosome 16 consistent with 16p11.2 microdeletion syndrome (Table 2).

**Table 2:** Diagnostic copy number variants (CNV).

ID	Sex	Age at seizure onset* (mos.)	Additional symptoms	Gene/Region	HGSVc** (if applicable)	CNV size	Copy number	Interpretation	Associated conditions
01	M	28	Ataxia, hemiparesis, clumsiness, speech delay	CACNA1A	c.(978+1_979–1)_(1082+1_1083–1)del	~242 bp	1	LP	Early infantile epileptic encephalopathy 42, Episodic ataxia, Familial hemiplegic migraine, Spinocerebellar ataxia 6
02	M	36	Prolonged neonatal jaundice, umbilical hernia, mild developmental and language delay, 5th finger clinodactyly	SLC6A8	c.1488_1899del	~766 bp	0	LP	Creatine deficiency syndrome
03	F	27	Microcephaly, hypotonia, dysmorphic, scoliosis, global developmental delay	1p36	N/A	2.1 Mb deletion	1	P	1p36 syndrome
04	M	24	Speech delay, query autism spectrum disorder	Angelman/Prader-Willi syndrome critical region	N/A	5.73 Mb deletion	1	P	Angelman syndrome/Prader-Willi syndrome
05	M	24	Global developmental delay		N/A	6.32 Mb deletion	1	P	
06	M	24	Language delay, repeated vomiting, weight loss, ataxia, abnormal MRI, abnormal EEG		N/A	4.87 Mb deletion	1	P	
07	F	28	Microcephaly, Hirschsprung, intellectual disability, dysmorphic, heart defect. Mowat-Wilson syndrome suspected	ZEB2 + 6 other OMIM genes (2 Morbid)	N/A	9 Mb deletion	1	P	Mowat-Wilson syndrome
08	F	24	Language delay	1q25.2-q44; Xq23-q28	N/A	72 Mb dup chr1q (786 genes); 40 Mb del chrXq (480 genes)	3 (chr1); 1 (chrX)	P	Patients with overlapping copy number variations have been reported in the DECIPHER database.
09	F	26	Language delay, motor impairment, abN MRI, abN EEG	MEF2C	N/A	122 kb deletion	1	P	Mental retardation
10	M	24	Developmental delay, progressive disease	PPT1	c.451C>T	Stop gained	N/A	P	Neuronal ceroid lipofuscinosis type I
					c.(124+1_125–1)_(234+1_235–1)del	~242 bp	1	LP	
11	M	54	Language delay, autism	16p11.2	N/A	772 kb deletion	1	P	16p11.2 microdeletion syndrome

M, male; F, female; LP, likely pathogenic; P, pathogenic; bp, base pair; kb, kilo base; Mb, mega base.

*unprovoked.

**NM transcripts: CACNA1A NM_001127221.1, SLC6A8 NM_005629.3.

In addition to the larger CNVs observed in 11 patients, we identified a clinically relevant single-nucleotide variant (SNV) or small deletion explaining the symptoms in 32 patients **(**Table 3). Interestingly, nine (21%) of the diagnosed patients had lysosomal storage diseases, specifically neuronal ceroid lipofuscinoses (NCLs). Six patients (14.0%; IDs 36–41) were diagnosed with neuronal ceroid lipofuscinosis type 2 (CLN2) caused by variants in *Tripeptidyl peptidase 1* (*TPP1)/(CLN2)*; five patients were homozygous for the c.622C>T p.(Arg208*) nonsense variant, while the sixth patient was homozygous for the c.533del p.(Pro178Glnfs*5) frameshift variant. The onset of seizures in the CLN2 cohort varied from 35 months to 44 months of age (Table 3), and genetic testing was carried out at 45 months to 56 months. All of these patients also had language delay and motor disturbances. One (2.3%) patient had a CNV and a nonsense variant in the *PPT1* gene (Table 2). Variants in this gene are associated with infantile, juvenile, and adult onset NCL (CLN1 disease). Lastly, in the lysosomal storage cohort, testing also identified two (4.7%; IDs 23 and 24) patients with pathogenic variants in *MFSD8* associated with neuronal ceroid lipofuscinosis type 7 (CLN7 disease). Both patients were male, and seizure onset was at 42 months age in one patient and 57 months in the other (Table 3).

**Table 3:** Diagnostic sequence variants.

ID	Sex	Age at seizure onset* (mos.)	Additional symptoms	Gene	HGSVc**	HGSVp	Variant type	gnomAD	Polyphen	SIFT	Mutaster	Inheritance/Zygosity	Interpretation	Associated conditions	ACMG evidence
12	F	25	Global developmental delay, frequent falls	CDKL5	c.473G>C	p.(Arg158Pro)	Missense	0/0	Probably damaging	Deleterious low confidence	Disease causing	XL/HET	LP	Angelman-like syndrome, Epileptic encephalopathy	PM1, PM2, PP3, PP5
13	M	36	Ataxia, language delay	CHD2	c.1126C>T	p.(Gln376*)	Stop gained	0/0	N/A	N/A	Disease causing	AD/HET	LP	Epileptic encephalopathy, childhood-onset	PVS1, PM2, PP5
14	F	35	Behaviour problems, irritability and feeding problems	CHD2	c.2190-1G>C	N/A	Splice acceptor	0/0	N/A	N/A	Disease causing	AD/HET	LP		PVS1, PM2, PP5
15	F	25	Motor delay, language delay, abnormal MRI	DCX	c.808+1G>A	N/A	Splice donor	0/0	N/A	N/A	N/A	XL/HET	LP	Lissencephaly, Subcortical laminal heterotopia	PVS1, PM2, PP5
16	F	32	Brain atrophy with demyelination	KCNA2	c.298C>T	p.(Arg100*)	Stop gained	0/0	N/A	N/A	Disease causing	AD/HET	LP	Early infantile epileptic encephalopathy	PVS1, PM2, PP5
17	F	25	Global developmental delay, no speech, truncal ataxic gait	KCNA2	c.1214C>T	p.(Pro405Leu)	Missense	0/0	Probably damaging	Deleterious	Disease causing	AD/HET	P		PM1, PM2, PP2, PP3, PP5
18	M	44	Developmental delay	KIF1A	c.37C>T	p.(Arg13Cys)	Missense	0/0	Probably damaging	Deleterious	Disease causing	AD/HET	LP	Mental retardation, Hereditary sensory neuropathy, Spastic paraplegia	PM1, PM2, PP5, PP3
19	F	34	Severe global developmental delay, generalized hypotonia, self-injurious behavior, high pain threshold	MECP2	c.2T>C	p.(Met1?)	Start lost	0/0	Unknown	N/A	Disease causing	XL/HET	LP	Rett syndrome, Angelman-like syndrome, Autism, Encephalopathy, Mental retardation,	PS1, PVS1, PM2, PP5
20	F	31	Global intellectual disability, ataxia, cerebral palsy	MECP2	c.509C>T	p.(Thr170Met)	Missense	0/0	Probably damaging	Deleterious	Disease causing	XL/HET	P		PP5, PM1, PM2, PM5, PP3
21	F	30	Developmental delay, sudden falls, gait disturbances	MECP2	c.916C>T	p.(Arg306Cys)	Missense	0/0	Probably damaging	Deleterious	Disease causing	XL/HET	P		PVS1, PP5, PM2, PS2,
22	F	42	Severe cognitive delay, autism spectrum disorder	MECP2	c.1157_1186delinsA	p.(Leu386Hisfs*9)	Frameshift	0/0	N/A	N/A	N/A	XL/HET	LP		PVS1, PM2, PP3
23	M	42	MRI suspicious for neuronal ceroid lipofuscinosis	MFSD8	c.881C>A	p.(Thr294Lys)	Missense	1/244390	Probably damaging	Deleterious	Disease causing	AR/HET	P	Neuronal ceroid lipofuscinosis type 7	PP5, PM2, PP2
					c.754+2T>A	N/A	Splice donor	3/276446	N/A	N/A	N/A	AR/HET	P		PVS1, PP5, PM2
24	M	57	Hearing loss, optic atrophy, ataxia	MFSD8	c.881C>A	p.(Thr294Lys)	Missense	1/244390	Possibly damaging	Deleterious	Disease causing	AR/HET	P		PP5, PM2, PP2
					c.754+2T>A	N/A	Splice	3/276446	N/A	N/A	Disease causing	AR/HET	P		PVS1, PP5, PM2
25	F	24	Motor difficulty, behavioral abnormalities, left parietal gliotic white matter lesion	PCDH19	c.85del	p.(Val29*)	Frameshift	0/0	N/A	N/A	N/A	XL/HET	LP	Early infantile epileptic encephalopathy	PVS1, PM2
26	F	27	Pontocerebellar atrophy, ataxia, generalized hypotonia, language and developmental delay, motor difficulty, visual impairment	PIGT	c.1582G>A	p.(Val528Met)	Missense	28/277070	Probably damaging	Deleterious	Disease causing	AR/HOM	P	Multiple congenital anomalies-hypotonia-seizures syndrome 3	PP5, PM2
27	F	28	Developmental delay	SCN1A	c.606C>A	p.(Tyr202*)	Stop gained	0/0	N/A	N/A	Disease causing	AD/HET	LP	Early infantile epileptic encephalopathy 6, Generalized epilepsy with febrile seizures plus, Familial febrile seizures 3A, Familial hemiplegic migraine	PVS1, PM2, PP5
28	M	24	Language delay, motor abnormalities, language delay, regression	SCN1A	c.2791C>T	p.(Arg931Cys)	Missense	0/0	Probably damaging	Deleterious	Disease causing	AD/HET	P		PM1, PP2, PM2, PM5, PP3, PP5
29	M	25	Developmental delay, autism spectrum, IgA deficiency	SCN1A	c.4547C>A	p.(Ser1516*)	Nonsense	0/0	N/A	N/A	Disease causing	AD/HET	P		PVS1, PM2, PP5
30	F	26	Developmental delay, abnormal myelination (supratentorial), low NAA/creatine in white matter	SCN2A	c.4514del	p.(Gly1505Valfs*24)	Frameshift	0/0	N/A	N/A	N/A	AD/HET	LP	Early infantile epileptic encephalopathy, Benign familial infantile seizures,	PVS1, PM2, PP5
31	F	26	Generalized hypotonia, language and developmental delay, motor difficulty, intellectual disability, abnormal MRI	SCN2A	c.5317G>A	p.(Ala1773Thr)	Missense	0/0	Probably damaging	Deleterious	Disease causing	AD/HET	LP		PM1, PP2, PM2, PM5, PP3, PP5
32	M	48	Developmental delay, speech delay, ataxia	SLC19A3	c.1264A>G	p.(Thr422Ala)	Missense	0/0	Probably damaging	Deleterious	Disease causing	AR/HOM	P	Thiamine metabolism dysfunction syndrome	PM1, PP3, PP5
33	F	24	Language delay, motor difficulty	STXBP1	c.624del	p.(Lys208Asnfs*24)	Frameshift	0/0	N/A	N/A	N/A	AD/HET	LP	Early infantile epileptic encephalopathy	PVS1, PM2, PP5
34	M	38	Delayed motor development, developmental and language regression after seizure onset, hypoplasia of frontal lobes, generalized hypomyelinization, mild leukoencephalopathy	STXBP1	c.246+1G>C	N/A	Splice donor	0/0	N/A	N/A	Disease causing	AD/HET	LP		PVS1, PM2, PP5
35	F	41	Language delay, motor impairments, behavioral abnormalities, sleep disturbances, visual impairment	SYNGAP1	c.2621_2625dupGCCAG	p.(Ser876Argfs*4)	Frameshift	0/0	N/A	N/A	N/A	AD/HET	LP	Mental retardation	PVS1, PM2, PP5
36	F	43	Delayed speech development, behavioral and sleep abnormalities, cerebellar atrophy	TPP1	c.622C>T	p.(Arg208*)	Nonsense	73/276920	N/A	N/A	Disease causing	AR/HOM	P	Neuronal ceroid lipofuscinosis type 2, Spinocerebellar ataxia	PVS1, PM2, PP5
37	M	36	Cerebellar and cerebral atrophy, language delay, motor difficulty, behavioral abnormalities	TPP1	c.622C>T	p.(Arg208*)	Nonsense	73/276920	N/A	N/A	Disease causing	AR/HOM	P		PVS1, PM2, PP5
38	F	35	Motor and language regression, ataxia, loosing ability to walk, choreoathetosis, enlargement of subarachnoid space, cerebral and cerebellar atrophy	TPP1	c.622C>T	p.(Arg208*)	Nonsense	73/276920	N/A	N/A	Disease causing	AR/HOM	P		PVS1, PM2, PP5
39	F	38	Hypotonia of right upper limb, delayed speech	TPP1	c.622C>T	p.(Arg208*)	Nonsense	73/276920	N/A	N/A	Disease causing	AR/HOM	P		PVS1, PM2, PP5
40	F	44	Speech delay, regression, ataxia, myoclonus	TPP1	c.533del	p.(Pro178Glnfs*5)	Frameshift	0/0	N/A	N/A	N/A	AR/HOM	LP		PVS1, PM2, PP5
41	F	36	Language delay, motor disturbance, cerebellar atrophy	TPP1	c.622C>T	p.(Arg208*)	Nonsense	73/276920	N/A	N/A	Disease causing	AR/HOM	P		PVS1, PM2, PP5
42	M	36	Developmental delay, regression, optic atrophy, abnormal MRI	RNASEH2B	c.554T>G	p.(Val185Gly)	Missense	0/0	probably damaging	deleterious	Disease causing	AR/HOM	P	Aicardi-Goutières syndrome	PM2, PP3, PM2, PP5
43	M	26	Mild global developmental delay, macrocephaly, hypopigmented macules, brain-cortical dysplasia	TSC1	c.1531dup	p.(Ser511Lysfs*24)	Frameshift	0/0	N/A	N/A	N/A	AD/HET	LP	Tuberous sclerosis	PVS1, PM2, PP5

M, male; F, female; LP, likely pathogenic; P, pathogenic; AD, autosomal dominant; AR, autosomal recessive; XL, X-linked; HET, heterozygous; HEM, hemizygous; HOM, homozygous.

*unprovoked.

**NM transcripts: CDKL5 NM_003159.2, CHD2 NM_001271.3, DCX NM_178153.2, KCNA2 NM_004974.3, KIF1A NM_004321.6, MECP2 NM_001110792.1, MFSD8 NM_152778.2, PCDH19 NM_001184880.1, PIGT NM_015937.5, SCN1A NM_001165963.1, SCN2A NM_021007.2, SLC19A3 NM_025243.3, STXBP1 NM_003165.3, SYNGAP1 NM_006772.2, TPP1 NM_000391.3, RNASEH2B NM_024570.3, TSC1 NM_00036.

Additionally, we identified four (9.3%; IDs 19–22) patients with *MECP2* variants associated with Rett syndrome (Table 3). Age of seizure onset varied from 30 to 42 months and all of these patients had developmental delay and motor disturbances. Three (7%; IDs 27–29) patients were found to have *SCN1A* variants; they experienced their first unprovoked seizures between the age of 24–28 months of life, and all had developmental delay. Detailed phenotypic information was not available for these patients, however, one of these patients was noted to have a provoked (febrile) seizure at 11 months of age. *KCNA2* variants were identified in two patients (IDs 30 & 31) whose first unprovoked seizures were at 25 and 32 months, respectively. In addition, both had developmental delay, ataxia, and MRI abnormalities. Two (4.7%; IDs 13 & 14) patients with seizure onset at 35 and 36 months who exhibited ataxia, developmental delay, feeding, and behavioral problems were found to have DEE-associated variants in *CHD2*. The cohort also included one (2.3%; ID 15) patient with lissencephaly caused by a heterozygous splice site variant in *DCX*. This patient’s first unprovoked seizure occurred at the age of 25 months and they had severe developmental delay and MRI abnormalities. One patient (ID 18) with a heterozygous *KIF1A* variant experienced their first unprovoked seizure at the age of 44 months and also had developmental delay. A heterozygous variant in *PCDH19* gene was identified in one female (ID 25) with MRI abnormalities, behavioral abnormalities, and motor difficulty. This patient’s first unprovoked seizure occurred at the age of 24 months. A homozygous missense variant was observed in *PIGT* in one patient (ID 26) with seizure onset at 27 months and with vision impairment, language delay, ataxia, and motor difficulty. Two patients (IDs 33 & 34) with *STXBP1* variants had their first unprovoked seizure at 24 and 35 months, respectively. In addition, both suffered from developmental delay. One patient (ID 43) with a *TSC1* duplication with seizure onset at 25 months also had mild global developmental delay, microcephaly, and hypopigmented macules. A four-nucleotide duplication was detected in *SYNGAP1* causing seizure onset at 41 months, vision impairment, motor impairment, language delay, and behavioral abnormalities (ID 35). A child (ID 42) with a homozygous missense variant in the Aicardi-Goutieres syndrome-associated gene, *RNASEH2B*, presented with optic atrophy, developmental delay, abnormal MRI and seizure onset at 36 months. Finally, a homozygous missense variant in *SLC19A3*, associated with biotin-thiamine-responsive basal ganglia disease, was identified. This patient (ID 32) suffered from developmental delay, ataxia, and seizure onset at 48 months (Table 3).

**Fig 2:**
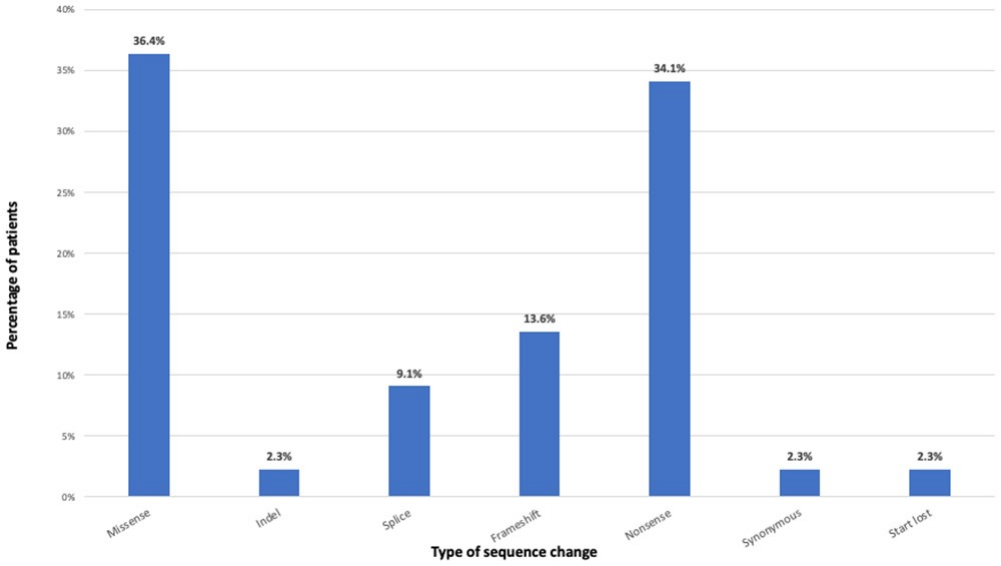
Type of likely pathogenic and pathogenic sequencing variants.

**Fig 3:**
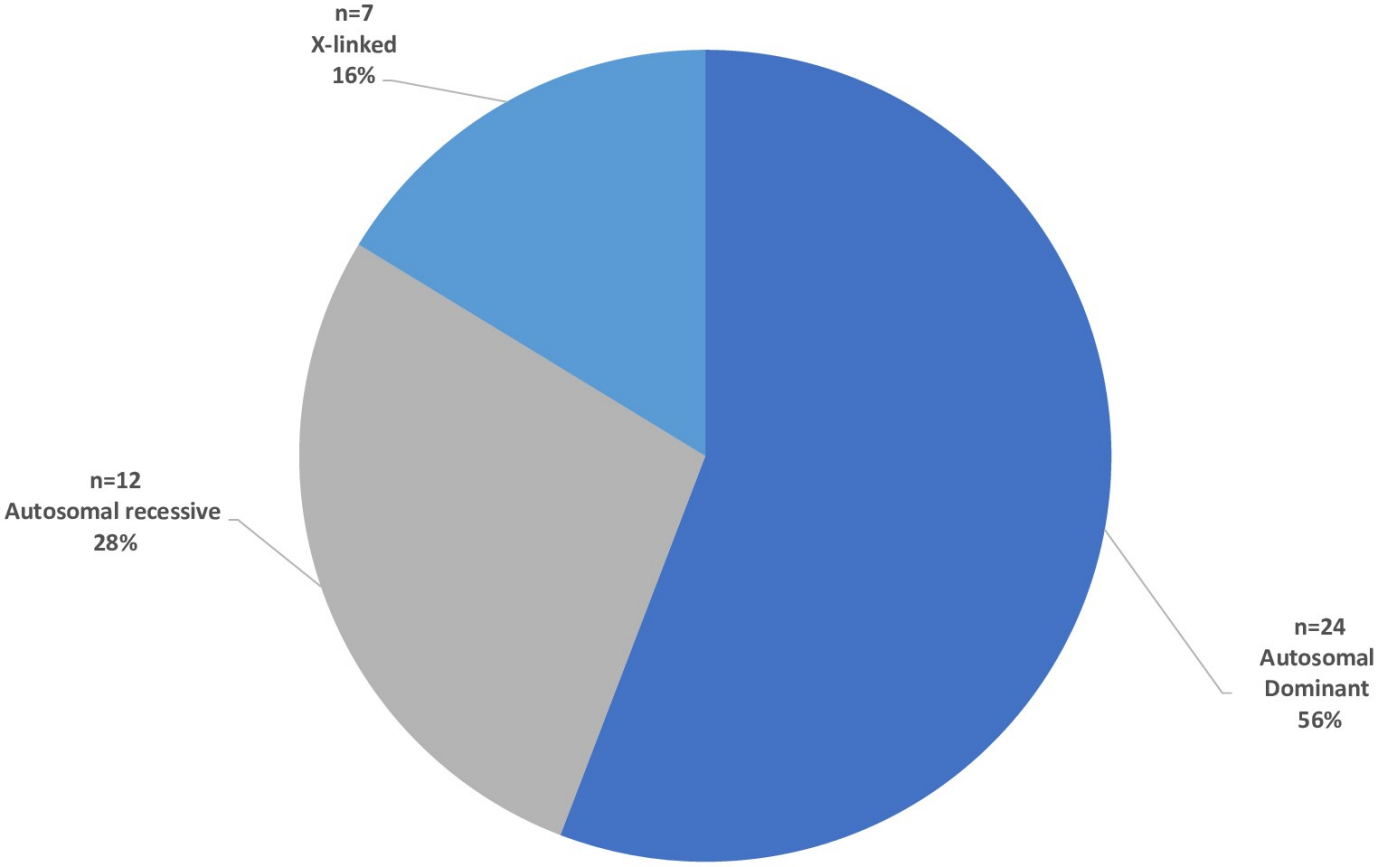
Mode of inheritance of diagnostic variants.

Among the 32 patients with SNVs or small deletions and duplications, missense variants were the most common variant type making up 38.4% of variants. Nonsense variants accounted for 34.1%, frameshift for 13.6% and splice site variants for 9.1% of all diagnostic variants (Fig 2). Indels and synonymous variants were both found with a frequency of 2.3%. Fifty-six percent of patients had variants consistent with autosomal dominant inheritance, 28% with autosomal recessive inheritance, and 16% with X-linked inheritance (Fig 3). Interestingly, 11 patients (25.6%) were diagnosed with an IEM; nine patients with NCL, one with creatinine deficiency syndrome (*SLC6A8*), and one with thiamine metabolism deficiency (*SLC19A3*). Among the patients with an IEM, the inheritance pattern was autosomal recessive in 91% and X-linked in 9%. This was markedly different among the non-IEM cases, where only 6.3% of variants were autosomal recessive, 75% were autosomal dominant, and 18.7% were X-linked. Disease-causing variants analyzed in this work were submitted to the ClinVar repository (SUB7819159).

In addition to the 43 patients with genetic diagnoses, our analysis revealed 17 additional patients (8.1%) with a highly suspicious VUS (Table 4). In these cases, the identified variants were novel and absent in reference databases, were predicted deleterious by bioinformatic predictions and showed a strong and specific correlation between the gene and the patient’s phenotype. The genes with a suspicious VUS were *ATRX*, *GABRA1 GRIA3*, *GRIN1*, *KCNA2*, *KCNQ2*, *KCNQ3*, *KDM5C*, *SCN1A*, *SCN8A*, *SLC6A1*, *SLC6A8*, *SLC9A6*, *STXBP1*, *UNC80*, and *ZEB2*. Further analyses, such as parental testing or functional assays, would be needed in order to reclassify the VUS detected in these genes to likely pathogenic. Interestingly, at least two patients (11.8%) within this group had variants in genes responsible for an IEM with associated neurological morbidity (*SLC6A1* and *SLC6A8*).

**Table 4:** Highly suspicious variants of uncertain significance (VUS).

ID	Sex	Age at seizure onset* (mos.)	Additional symptoms	Gene	HGSVc**	HGSVp	Variant type	gnomAD	Polyphen	SIFT	Mutaster	Inheritance/Zygosity	Associated conditions	ACMG evidence
44	M	40	Global developmental, microcephaly	ATRX	c.7396G>A	p.(Gly2466Ser)	Missense	0/0	Probably damaging	Deleterious	Disease causing	XL/HEM	Alpha-thalassemia/intellectual disability syndrome	PM2, PP2, PP3
45	M	27	Normal development, seizures in sleep, hyperintense signal in dorsal part of brainstem	GABRA1	c.406G>T	p.(Ala136Ser)	Missense	0/0	Probably damaging	Tolerated	Disease causing	AD/HET	Childhood absence epilepsy, Juvenile myoclonic epilepsy, Early infantile epileptic encephalopathy	PM2, PP2, PP3
46	M	24	Delayed development, dizziness	GRIA3	c.1648C>T	p.(Pro550Ser)	Missense	0/0	Probably damaging	Deleterious	Disease causing	XL/HEM	Mental retardation	PM2, PP2, PP3
47	M	25	Dysmorphic features, dysarthria, ataxia, oculomotor apraxia, failure to thrive, psychomotor developmental delay, no speech, delayed myelination, signal hyperintensity of globus pallidus, hypotonia, hypomelanotic mark (1), cafe au lait spot (1), Mongolian spots (2)	KCNA2	c.1222G>C	p.(Val408Leu)	Missense	0/0	Probably damaging	Deleterious	Disease causing	AD/HET	Early infantile epileptic encephalopathy	PM2, PM5, PP3
48	M	24	Language delay	KCNQ2	c.827C>A	p.(Thr276Asn)	Missense	0/0	Probably damaging	Tolerated	Disease causing	AD/HET	Benign familial neonatal seizures, Early infantile epileptic encephalopathy, Myokymia	PM2, PM5, PP3
49	F	40	Language delay, motor difficulty	KCNQ3	c.2533T>A	p.(Phe845Ile)	Missense	0/0	Probably damaging	Deleterious	Disease causing	AD/HET	Benign neonatal seizures	PM2
50	M	38	Language delay, cognitive delay	KCNQ3	c.1289T>C	p.(Val430Ala)	Missense	0/0	Probably damaging	Tolerated	Disease causing	AD/HET		PM2, PP3
51	M	30	Developmental delay, language delay	KDM5C	c.1354G>A	p.(Gly452Ser)	Missense	0/0	Probably damaging	Deleterious	Disease causing	XL/HEM	Mental retardation syndromic Claes-Jensen	PM2, PP2, PP3
52	M	52	Language delay, motor delay, behaviour abnormalities, sleep disturbances	GRIN1	c.2144C>T	p.(Ala715Val)	Missense	0/0	Probably damaging	Tolerated	Disease causing	AD, AR/HET	Autosomal dominant mental retardation 8	PM2, PP2, PP3
53	M	30	Language delay, motor decline	SCN1A	c.5797C>T	p.(Arg1933*)	Stop gained	0/0	N/A	N/A	Disease causing	AD/HET	Early infantile epileptic encephalopathy	PM2, PP3
54	F	48	Mild coordination problems	SCN8A	c.4419+2_4419+3del		Splice donor	0/0	N/A	N/A	N/A	AD/HET	Cognitive impairment, Early infantile epileptic encephalopathy	PM2, PP3
55	M	40	Developmental delay, autistic behaviour	SLC6A1	c.1178G>A	p.(Gly393Asp)	Missense	0/0	Probably damaging	Deleterious	Disease causing	AD/HET	Myoclonic-astastic epilepsy	PM1, PM2, PP3
56	M	24	Moderate global developmental delay, autistic behaviors	SLC6A8	c.428_430delACT	p.(Tyr143del)	Inframe deletion	0/0	N/A	N/A	N/A	XL/HEM	Creatine deficiency syndrome	PM2, PM4, PP3
57	M	26	Ataxic gait, language delay, behavioral disturbance, ADHD, hirsutism, inverted nipples, hypogenesis of the corpus callosum, diffuse hypomyelinization, hypogonadism, osteosarcoma, pes planus, genu valgum	SLC9A6	c.1474G>A	p.(Ala492Thr)	Missense	0/0	Probably damaging	Deleterious	Disease causing	XL/HEM	Mental retardation, syndromic, Christianson	PM1, PM2, PP3, BP1
58	F	36	Fine motor impairment, speech delay, intellectual disability, autistic features	STXBP1	c.1758T>G	p.(Asn586Lys)	Missense	0/0	Probably damaging	Tolerated	Disease causing	AD/HET	Early infantile epileptic encephalopathy	PM2, PP2, BP4
59	M	25	Significant language delay	UNC80	c.3883G>C	p.(Glu1295Gln)	Missense	28/174746	Benign	Deleterious	Disease causing	AR/HOM	Infantile hypotonia with psychomotor retardation and characteristic facies 2	PM2, BP1
60	M	41	Speech delay, hyperactivity, consanguineous parents	ZEB2	c.807+5G>A	N/A	Splice	0/0	N/A	N/A	N/A	AD/HET	Mowat-Wilson syndrome	PM2

M, male; F, female; LP, likely pathogenic; P, pathogenic; AD, autosomal dominant; AR, autosomal recessive; XL, X-linked; HET, heterozygous; HEM, hemizygous; HOM, homozygous.

*unprovoked.

**NM transcripts: ATRX NM_000489.4, GABRA1 NM_000806.5, GRIA3 NM_000828.4, KCNA2 NM_004974.3, KCNQ2 NM_172107.2, KCNQ3 NM_004519.3, KDM5C NM_004187.3, GRIN1 NM_007327.3, SCN1A NM_001165963.1, SCN8A NM_014191.3, SLC6A1 NM_003042.3, SLC6A8 NM_005629.3, SLC9A6 NM_001042537.1, STXBP1 NM_003165.3, UNC80 NM_032504.1.

In this cohort, none of the patients who received a diagnosis were found to have one or more of the custom-added 70 non-coding/deep intronic variants associated with epilepsy.

## Discussion

To our knowledge, this represents one of the first studies in patients with epilepsy onset between 24 to 60 months of life with comprehensive genetic testing [26]. The inclusion criteria were selected according to common early findings of CLN2 disease: unprovoked seizure after 2 years of age and at least one of motor disturbance, speech delay, MRI abnormalities and abnormal EEG. Indeed, the group of patients molecularly diagnosed with this panel mostly presented with motor disturbance (79.1%) and developmental delay (65.1%). In this patient cohort, we demonstrated a molecular diagnostic yield of 20.4% [21], which is higher than the 4–14% presented in previous studies of patients with epilepsy onset after two years of age [8,18]. One of the reasons for the difference could be the characteristics of the cohort. In this study, only patients with seizures and at least one additional neurological finding were included and thus this cohort may be more severely affected. Further, patients in the Moller et al [8] study ranged in age from 2 years– 9 years. As the likelihood of a genetic diagnosis decreases with increasing age of seizure oneset, this may also contribute to the difference in diagnoses. The diagnostic yield may also be affected by the quality and performance of the sequencing and bioinformatics pipeline in addition to the number of genes included in the analysis. In our study, 186 (88.2%) of the patients were analyzed with a panel of 283 genes while 25 (11.8%) were analyzed with a 195-gene panel. Both panels had >20x coverage in >99.5% of target nucleotides. In previous studies, the panel size varied from 29 to 102 genes and the quality metrics indicated that 3–5% of target regions were not covered [8,18]. These metrics represent a significant difference between studies and are likely to contribute to the differences in yield. In addition, several genes that yielded diagnostic results in our study, such as the NCL genes, are not commonly covered by standard epilepsy panels and were also missing from the analyses by Moller et al. and Oates et al [8,18].

A panel testing approach was selected for this study as we wanted to evaluate the clinical utility and diagnostic yield of panel-based testing within this age group. While we do acknowledge that a high-quality WES testing strategy may have increased the diagnostic yield in this cohort, a panel-based approach remains the standard of care for the evaluation of patients with epilepsy.

A significant contributor to yield was the high prevalence of CNVs among diagnosed patients. In this study, >5% of all patients and 25.6% of diagnosed patients harbored a clinically significant CNV. Small intragenic deletions represented 27.2% of all CNVs. Several studies have previously addressed the role of CNVs in patients with epilepsy. Mefford et al. [27] used a genome wide array CGH with average probe spanning 38kb to study a cohort of 517 mixed type epilepsies. They identified a rare, potentially causative CNV in 8.9% of tested patients. That study was published in 2010 and thus lacked access to reference databases to assist in interpretation of identified CNVs, and the technology utilized did not have adequate performance for the detection of smaller intragenic CNVs [23]. Niestroj *et al* [28] studied a cohort of >10,000 subjects with one of four different epilepsy phenotypes with a genome wide SNP array and identified a likely pathogenic CNV is 1.15%-2.88% of patients, depending on the epilepsy phenotype. The authors point out that especially small structural variants were not detectable with the available genotyping platform. A recent study of >8500 patients with epilepsy used an exon-level array CGH with enhanced performance of 70 selected epilepsy genes [10]. The authors reported that <2% of epilepsy patients carried a clinically relevant CNV, contributing to 10% of diagnosed cases [10]. Strikingly, approximately 60% of detected CNVs were intragenic with one- to two-exon CNVs (37.0%).

The detection of CNVs has shifted towards molecular genetic technologies given the recent advances in techniques. While conventionally available chromosome microarrays and SNP arrays have CNV detection capabilities of ~20kb– 200kb, whole exome sequencing has been reported to have CNV detection capabilities of 100bp—~150kb [29]. The assays used in this study were validated to detect single exon deletions with a sensitivity of 71.5% (NextSeq OS-Seq assay) and 92.3% (NovaSeq WES assay) and detected CNVs ranging in size from 242bp—>70Mbp. The application of molecular techniques for the detection of CNVs has the potential to identify both exon-level CNVs in addition to large microdeletions and microduplications, making it a powerful tool for the simultaneous evaluation of sequence variants and CNVs.

Despite the significant difference in the patient cohorts and technologies used, our study, in addition to the Lindy *et al* study [10], strongly support the role of incorporating high-resolution, molecular-based CNV detection in the genetic testing of patients with epilepsy. Considering the high prevalence of small intragenic CNVs, typical and commonly used chromosomal array CGH tests may not be sufficient for epilepsy patients. Laboratories that put a high premium on sequencing coverage, sequencing uniformity, and bioinformatic algorithm development can offer similar, or better, CNV detection than CGH arrays [30].

Research and innovations in the field of rare diseases, including epilepsies, are improving the clinical utility of genetic testing. A genetic diagnosis can, in many cases, be used for targeting and optimizing therapy. Moreover, the declining cost of sequencing (80–90% over the past 10 years) have increased the use of genetic testing in diagnosing patients [31]. In our study, one of the striking findings was the high prevalence of CLN2 disease cases among these patients (14% of diagnosed patients). This is especially interesting since a targeted enzyme replace therapy, cerliponase alfa, has been approved by FDA and EMA to treat patients with CLN2 disease [32]. The treatment, by intraventricular infusion of cerliponase alfa in patients with CLN2, resulted in less decline in motor and language function than in historical controls [32]. CLN2 disease is caused by deficiency of the lysosomal enzyme TPP1 and is characterized by rapid psychomotor decline and epilepsy. Seizures are one of the first symptoms noted in patients with CLN2 disease typically occurring after two years of age (median age 35 months), often preceded by history of language delay. Subsequently, substantial loss of language and motor skills happen by the age of four to five years and death between six and twelve years of age. Unfortunately, due to low awareness of the disease, non-specific early signs and limited access to specific testing in some regions [33,34] the diagnosis of CLN2 disease is usually reached late at a mean age of five years (median age 54 months). Remarkably, patients with CLN2 disease in this study were diagnosed at a mean age of 38.7 months with a mean time from seizure onset to diagnosis of 10 months, a significantly shorter time compared to natural history diagnostic data [34], demonstrating that use of a comprehensive epilepsy gene panel can effectively and timely diagnose patients with CLN2 disease. Interestingly, consistent with our study, three other reports have recently shown *TPP1* (*CLN2*) pathogenic or likely pathogenic variants and CLN2 diagnosis being a relatively common finding when using NGS panels for diagnostics of pediatric seizure patients [10,26]. These findings stress the importance of the genes included in the selected panel when evaluating the diagnostic yield and appropriateness of the panel.

Other treatable disorders within our cohort included biotin-thiamine-responsive basal ganglia disease caused by variants in *SLC19A3* (Table 3). Without biotin and thiamine replacement therapy, the outcome for these patients can be lethal [35]. Recently, the targeted therapy everolimus was approved for the treatment of *TSC1* related disease [36]. *SLC6A8* creatinine transporter deficiency can be effectively treated with L-arginine, glycine and creatinine supplements [37]. In addition, the EMA and FDA have approved stiripentol and cannabidiol for the treatment of Dravet syndrome, mainly caused by variants in the *SCN1A* gene [38–40]. Moreover, it has been suggested that patients with Dravet syndrome and patients with *SCN1A* variants causing loss of function of the Na^+^ channel should avoid Na^+^ channel blocking anti-epileptic drugs. Studies on existing anti-epileptic medications have provided evidence on the optimal treatment combinations for epileptic disorders related to *PCDH19* and *STXBP1* [31–34].

In addition to existing targeted management strategies, there were several diagnoses with ongoing interventional clinical trials. These included disorders caused by variants in *CACNA1A*, *CDKL5*, *MECP2*, and *SYGNAP1*. In brief, at least 27 (63%) patients were diagnosed with a disorder for which there is targeted treatment, evidence for optimizing pharmaceutical treatment, or on-going clinical trials available (S3 Table).

A potential limitation of the study is that the patients were referred to us as part of a no-cost testing program. The program had strict eligibility criteria, which were outlined on the requisition form, however, patients were assessed and referred for testing by their own health care provider. Referring physicians were required to sign a statement confirming that their patient met the eligibility criteria but systematic evaluation by a pediatric neurologist or neuroradiologist was not required. Misapplication of this no-cost testing program could lead to atypical or misleading correlations between genotype and phenotype. One area of interest was the age of the first unprovoked seizure. For most of the molecular diagnoses, the age of seizure onset between two and five years is consistent with the described disease phenotype. However, in some cases, the findings may suggest a novel or rare/atypical form of the disease. Variants in *CDKL5* are typically associated with early onset epileptic encephalopathy with seizures usually starting between three and six months of age [30,35,36]. According to the referral, our *CDKL5* patient had her first unprovoked seizure at the age of 25 months, which represents an atypical age of onset (Table 3). One patient with a homozygous *PIGT* variant had their first unprovoked seizure at the age of 27 months (Table 3), which is later in onset compared to the published cases with *PIGT*-related multiple congenital anomalies hypotonia-seizure syndrome 3 (MCAHS3) [41]. Interestingly, our cohort included three *SCN1A* cases and two *SCNA2* cases (Table 3). The reported age of unprovoked seizure onset varied from 24 to 28 months. One of the patients with an *SCN1A* variant was reported to have their first provoked (febrile) seizure at 11 months of age, consistent with the natural history of SCN1A-related seizures [38–40]. Detailed medical records regarding the other two patients were not available to the authors so it is not clear if they had provoked seizures prior to 24 months of age or if they represent a broadening of they phenotype associated with *SCN1A* variants. Our findings support the hypothesis that genes classically associated with early onset epilepsy should be included as part of the routine genetic diagnostic process in children with onset of seizures after two years of age, and especially when seizure symptoms are associated with other neurological abnormalities.

A further limitation of this study was the lack of segregation analysis. Variants classified as pathogenic or likely pathogenic had sufficient evidence for classification without parental testing. However, 17 patients (8.1%) in this cohort were found to have a highly suspicious VUS. Testing of the parents in these patients would have likely been sufficient for classification as likely pathogenic, resulting in a significant addition to diagnostic yield. Thus, when possible, testing of parents should be considered to resolve the classification of suspicious VUS.

In conclusion, in children with epilepsy who had their first unprovoked seizure at 24 to 60 months of age and at least one other neurological finding, NGS testing demonstrated a diagnostic yield of 20.4%. Our findings further the importance of the early use of genetic testing, including high-resolution CNV detection, in this age group to efficiently identify severe disorders with targeted management available such as CLN2. Considering the high prevalence of CLN2, this study supports the addition of *TPP1* (*CLN2*) and other genes linked to NCLs in diagnostic NGS-based epilepsy panels.

## Supporting information

S1 Table Gene content of panels used in the analysis. (PDF)

S2 Table Non-coding variants that are covered by the NGS analysis. (PDF)

S3 Table Next best action after molecular diagnosis. (PDF)
