# Supplementary material for: Next-generation sequencing in childhood-onset epilepsies: Diagnostic yield and impact on neuronal ceroid lipofuscinosis type 2 (CLN2) disease diagnosis
Source: PLoS One. 2021 Sep 1;16(9):e0255933. doi: 10.1371/journal.pone.0255933 (PMC8409681; doi:10.1371/journal.pone.0255933)
Supplement: S1 Table — (PDF) [file pone.0255933.s001.pdf]

Supplemental Table 1 Gene content of panels used in the analysis. Genes added to the second panel are highlighted in gray.

Panel	Genes
194 genes	ABCD1, ADAR, ADSL, AFG3L2, AGA, AIMP1, ALDH5A1, ALDH7A1, ALG13, AMACR, AMT, ARG1, ARHGEF9, ARSA, ARX, ASAH1, ASPA, ATP13A2, ATRX, BTD, CACNA1A, CACNA1H, CACNB4, CASK, CASR, CDKL5, CERS1, CHD2, CHRNA2, CHRNA4, CHRNB2, CLCN2, CLN3, CLN5, CLN6, CLN8, CNTNAP2, COL4A1, COX15, CPT2, CSF1R, CSTB, CTSD, CTSF, CUL4B, DARS2, DCX, DEPDC5, DNAJC5, DNM1, DOCK7, DPYD, EARS2, EEF1A2, EFHC1, EIF2B1, EIF2B2, EIF2B3, EIF2B4, EIF2B5, EPM2A, ETFA, ETFB, ETFDH, FAM126A, FH, FLNA, FOLR1, FOXG1, FOXRED1, GABRA1, GABRB3, GABRG2, GALC, GAMT, GCDH, GCH1, GFAP, GJC2, GLDC, GNAO1, GNE, GOSR2, GPHN, GRIA3, GRIN2A, GRIN2B, GRN, HCN1, HEPACAM, HNRNPU, HSD17B10, HSPD1, IQSEC2, KCNA1, KCNA2, KCNB1, KCNC1, KCNQ2, KCNQ3, KCNT1, KCTD7, KDM5C, KIF1A, L2HGDH, LGI1, MARS2, MBD5, MECP2, MED12, MEF2C, MFSD8, MLC1, MOCS1, MTHFR, MTOR, NDUFAF5, NECAP1, NEU1, NHLRC1, NOTCH3, NRXN1, OFD1, OPHN1, PCDH19, PGK1, PHF6, PIGA, PLCB1, PLP1, PNKP, PNPO, POLG, POLR3A, POLR3B, PPT1, PRICKLE1, PRICKLE2, PRODH, PRRT2, PSAP, PTS, PURA, QDPR, RAB39B, RELN, RNASEH2A, RNASEH2B, RNASEH2C, RNASET2, SAMHD1, SCARB2, SCN1A, SCN1B, SCN2A, SCN8A, SCN9A, SERPINI1, SIK1, SLC2A1, SLC6A1, SLC6A8, SLC9A6, SLC12A5, SLC13A5, SLC19A3, SLC25A15, SLC25A22, SLC35A2, SLC46A1, SMS, SNAP25, SOX10, SPTAN1, ST3GAL3, ST3GAL5, STX1B, STXBP1, SUMF1, SUOX, SYN1, SYNGAP1, SZT2, TBC1D24, TCF4, TPP1, TREX1, TSC1, TSC2, TUBB4A, UBE2A, UBE3A, WDR45, WWOX and ZEB2
283 genes	ABAT, ABCD1, ADAR, ADSL, AFG3L2, AGA, AIFM1, AIMP1, ALDH3A2, ALDH5A1, ALDH7A1, ALG13, AMACR, AMT, AP4B1, AP4E1, AP4M1, AP4S1, APOPT1, ARG1, ARHGEF9, ARSA, ARX, ASAH1, ASNS, ASPA, ATP1A3, ATP13A2, ATRX, BRAT1, BTD, CACNA1A, CACNA1H, CACNB4, CASK, CASR, CC2D1A, CDKL5, CERS1, CHD2, CHRNA2, CHRNA4, CHRNB2, CLCN2, CLCN4, CLN3, CLN5, CLN6, CLN8, CNTNAP2, COL4A1, COX6B1, COX15, CPT2, CSF1R, CSTB, CTC1, CTSD, CTSF, CUL4B, CYP27A1, D2HGDH, DARS, DARS2, DCX, DDC, DEPDC5, DHFR, DNAJC5, DNM1, DNM1L, DOCK7, DPYD, DPYS, EARS2, ECHS1, ECM1, EEF1A2, EFHC1, EIF2B1, EIF2B2, EIF2B3, EIF2B4, EIF2B5, EPM2A, ETFA, ETFB, ETFDH, ETHE1, FA2H, FAM126A, FAR1, FARS2, FGF12, FH, FLNA, FOLR1, FOXG1, FOXRED1, GABRA1, GABRB2, GABRB3, GABRG2, GALC, GAMT, GCDH, GCH1, GFAP, GFM1, GJC2, GLB1, GLDC, GLRB, GNAO1, GNB1, GNE, GOSR2, GPHN, GRIA3, GRIK2, GRIN1, GRIN2A, GRIN2B, GRN, GTPBP3, HACE1, HCN1, HECW2, HEPACAM, HIBCH, HNRNPU, HSD17B10, HSPD1, HTRA1, HTT, IBA57, IQSEC2, KCNA1, KCNA2, KCNB1, KCNC1, KCNH1, KCNQ2, KCNQ3, KCNT1, KCTD7, KDM5C, KIF1A, L2HGDH, LGI1, LMNB1, LRPPRC, LYRM7#, MAGI2, MARS2, MBD5, MECP2, MED12, MEF2C, MFSD8, MLC1, MOCS1, MRPL44, MTFMT, MTHFR, MTOR, NACC1, NDUFAF5, NDUFAF6, NDUFS2, NDUFS4, NDUFS7, NDUFS8, NDUFV1, NECAP1, NEU1, NFU1, NHLRC1, NOTCH3, NRXN1, NUBPL, OFD1, OPHN1, PCDH19, PGK1, PHF6, PIGA, PIGN, PIGO, PIGT, PIGV, PLCB1, PLP1, PNKP, PNPO, POLG, POLR3A, POLR3B, PPT1, PRICKLE1, PRIMA1, PRODH, PRRT2, PSAP, PTS, PURA, PYCR2#, QDPR, RAB39B, RARS, RELN, RMND1, RNASEH2A, RNASEH2B, RNASEH2C, RNASET2, RNF216, ROGDI, SAMHD1, SCARB2, SCN1A, SCN1B, SCN2A, SCN8A, SCN9A, SCO1, SDHAF1, SERAC1, SERPINI1, SIK1, SLC2A1, SLC6A1, SLC6A8, SLC9A6, SLC12A5, SLC13A5, SLC19A3, SLC25A1, SLC25A15, SLC25A22, SLC35A2, SLC39A8, SLC46A1, SMS, SNAP25, SNORD118, SOX10, SPATA5, SPTAN1, ST3GAL3, ST3GAL5, STX1B, STXBP1, SUMF1, SUOX, SYN1, SYNGAP1, SYNJ1, SZT2, TAF1, TBC1D24, TBCD, TBCE, TBCK, TBL1XR1, TCF4, TPP1, TREX1, TSC1, TSC2, TTC19, TUBB4A, UBA5, UBE2A, UBE3A, UNC80, VPS13A, WDR26, WDR45, WWOX, YY1, ZEB2 and ZFYVE26.
