# Supplementary material for: Next-generation sequencing in childhood-onset epilepsies: Diagnostic yield and impact on neuronal ceroid lipofuscinosis type 2 (CLN2) disease diagnosis
Source: PLoS One. 2021 Sep 1;16(9):e0255933. doi: 10.1371/journal.pone.0255933 (PMC8409681; doi:10.1371/journal.pone.0255933)
Supplement: S2 Table — (PDF) [file pone.0255933.s002.pdf]

Supplemental Table 2
Non-coding variants that are covered by the NGS analysis

Gene	Position	Nucleotide change	Transcript
ADSL	Chr22:40742514	c.-49T>C	NM_000026.2
ALDH3A2	Chr17:19561044	c.681-14T>A/G	NM_001031806.1
ALDH7A1	Chr5:125907053	c.696-502G>C	NM_001182.4
AMT	Chr3:49459938	c.-55C>T	NM_000481.3
ARG1	Chr6:131901748	c.306-611T>C	NM_000045.3
ARSA	Chr22:51064121	c.1108-12C>G	NM_000487.5
BTD	Chr3:15687154	c.*159G>A	NM_000060.2
CACNA1A	Chr19:13341036	c.5404-13G>A	NM_001127221.1
CASR	Chr3:121994640	c.1378-19A>C	NM_001178065.1
CDKL5	ChrX:18525053	c.-162-2A>G	NM_003159.2
CLN3	Chr16:28497984	c.461-13G>C	NM_000086.2
COL4A1	Chr13:110802675	c.*35C>A	NM_001845.4
COL4A1	Chr13:110802678	c.*32G>T	NM_001845.4
COL4A1	Chr13:110802679	c.*31G>T	NM_001845.4
D2HGDH	Chr2:242680425	c.293-23A>G	NM_152783.3
DARS2	Chr1:173797449	c.228-21_228-20insC	NM_018122.4
EIF2B5	Chr3:183855941	c.685-13C>G	NM_003907.2
ETFDH	Chr4:159593534	c.-75A>G	NM_004453.2
FGF12	Chr3:191857076	c.*4722T>C	NM_021032.4
GABRA1	Chr5:161274418	c.-248+1G>T	NM_000806.5
GABRB3	Chr15:27020313	c.-2204G>A	NM_000814.5
GABRB3	Chr15:27020399	c.-2290T>C	NM_000814.5
GALC	Chr14:88459917	c.-74T>A	NM_001201402.1
GAMT	Chr19:1399508	c.391+15G>T	NM_138924.2
GCDH	Chr19:13010271	c.1244-11A>G	NM_000159.3
GJC2	Chr1:228337558	c.-170A>G	NM_020435.3
GJC2	Chr1:228337561	c.-167A>G	NM_020435.3
GJC2	Chr1:228337709	c.-20+1G>C	NM_020435.3
GRN	Chr17:42422701	c.-9A>G	NM_002087.2
GRN	Chr17:42422705	c.-8+3A>T	NM_002087.2
GRN	Chr17:42422707	c.-8+5G>C	NM_002087.2

L2HGDH	Chr14:50735527	c.906+354G>A	NM_024884.2
MOCS1	Chr6:39874534	c.*365_*366delAG	NM_005943.5
MTHFR	Chr1:11850973	c.1753-18G>A	NM_005957.4
MTHFR	Chr1:11863212	c.-13-28_-13-27delCT	NM_005957.4
NDUFAF6	Chr8:96046914	c.298-768T>C	NM_152416.3
NDUFS7	Chr19:1386643	c.17-1167C>G	NM_024407.4
NUBPL	Chr14:32319298	c.815-27T>C	NM_025152.2
OFD1	ChrX:13768358	c.935+706A>G	NM_003611.2
OFD1	ChrX:13773245	c.1130-22_1130-19delAATT	NM_003611.2
PLP1	ChrX:103042405	c.454-322G>A	NM_000533.3
PLP1	ChrX:103042413	c.454-314T>A/G	NM_000533.3
PNKP	Chr19:50364799	c.1387-33_1386+49delCCTCCTCCCCTGACCCC	NM_007254.3
POLR3A	Chr10:79769273	c.1909+22G>A	NM_007055.3
POLR3A	Chr10:79769277	c.1909+18G>A	NM_007055.3
POLR3B	Chr12:106804589	c.967-15A>G	NM_018082.5
POLR3B	Chr12:106831447	c.1857-12A>G	NM_018082.5
PPT1	Chr1:40539203	c.*526_*529delATCA	NM_000310.3
PPT1	Chr1:40558194	c.125-15T>G	NM_000310.3
PSAP	Chr10:73583679	c.778-26C>A	NM_001042465.1
PTS	Chr11:112098994	c.84-323A>T	NM_000317.2
PTS	Chr11:112099026	c.84-291A>G	NM_000317.2
PTS	Chr11:112100215	c.164-716A>T	NM_000317.2
QDPR	Chr4:17500790	c.436+2552A>G	NM_000320.2
RNASEH2B	Chr13:51501530	c.65-13G>A	NM_024570.3
SCN1A	Chr2:166848946	c.4820-14T>G	NM_006920.4
SCN1A	Chr2:166854699	c.4306-14T>G	NM_006920.4
SCN1A	Chr2:166908215	c.964+14T>G	NM_006920.4
SCN1A	Chr2:166913031	c.384-21T>A	NM_006920.4
SLC19A3	Chr2:228560811	c.980-14A>G	NM_025243.3
SLC2A1	Chr1:43395462	c.680-11G>A	NM_006516.2
SOX10	Chr22:38379877	c.-84-2A>T	NM_006941.3
SOX10	Chr22:38412215	c.-31954C>T	NM_006941.3
TBCD	Chr17:80851411	c.1564-12C>G	NM_005993.4

TPP1	Chr11:6637752	c.887-18A>G	NM_000391.3
TSC2	Chr16:2098067	c.-30+1G>C	NM_000548.3
TSC2	Chr16:2110656	c.976-15G>A	NM_000548.3
TSC2	Chr16:2127477	c.2838-122G>A	NM_000548.3
TSC2	Chr16:2138031	c.5069-18A>G	NM_000548.3
ZEB2	Chr2:145274987	c.-69-1G>A	NM_014795.3
