# Supplementary material for: Next-generation sequencing in childhood-onset epilepsies: Diagnostic yield and impact on neuronal ceroid lipofuscinosis type 2 (CLN2) disease diagnosis
Source: PLoS One. 2021 Sep 1;16(9):e0255933. doi: 10.1371/journal.pone.0255933 (PMC8409681; doi:10.1371/journal.pone.0255933)
Supplement: S3 Table — (PDF) [file pone.0255933.s003.pdf]

Supplemental Table 3. Next best action after molecular diagnosis

Gene	Condition	Possible Management Implication
TPP1	Neuronal Ceroid Lipofuscinosis type 2	Ceriponase alfa approved ERT therapy for eligible patients in the EU, US, Ukraine, Brazil, Australia, Canada, Mexico[2,3]
MECP2	Rett syndrome	Interventional clinical trials [1,5,6]
KCNA2	Early infantile epileptic encephalopathy	No management implication
CDKL5	Angelman-like syndrome, Early infantile epileptic encephalopathy-2	Ataluren for nonsense variants (clinical trial) [4]<br>Ganaloxone (clinical trial) [4]
SCN2A	Early infantile epileptic encephalopathy Epileptic encephalopathy, early infantile, Seizures, benign familial infantile	No disease-altering treatment, Specific AEDs are recommended according to the variant (depending on gain or loss of function)[10], Observational clinical trial[9], Interventional clinical trial[5]
MFSD8	Neuronal Ceroid Lipofuscinosis type 7	No management implication, Observational clinical trial[20]
SCN1A	Dravet Syndrome	Avoid sodium channel blockers[8]<br>Interventional clinical trials open for enrolment [4,5] EPIDIOLEX (cannabidiol) approved treatment [4,5]
CHD2	Epileptic Encephalopathy childhood onset	No management implication, Observational clinical trial[22]
STXBP1	Epileptic encephalopathy early infantile	Pharmacogenomic information available (AEDs to consider phenobarbital, valproic acid, and vigabatrin[16]), Observational clinical trial[2]
UBE3A	Angelman syndrome, Prader–Willi syndrome	Interventional clinical trials [23,24]
CACNA1A	Epileptic encephalopathy early infantile 42, Episodic ataxia, Migraine familial hemiplegic, Spinocerebellar ataxia 6	No management implication
TSC1	Tuberous sclerosis	Interventional clinical trials [25,26]<br>Everolimus approved treatment (mTOR inhibitors)[25]
SYNGAP1	Mental retardation	Observational clinical trial[27]
72-kb del. 16p11.2	16p11.2 microdeletion syndrome	Observational and interventional clinical trials[28,29]<br>Specific deficit target treatment[17]<br>Recommendation: to establish neurodevelopement[17]
SLC19A3	Thiamine metabolism dysfunction syndrome	Biotin and thiamine therapy[11, 18]

PIGT	Multiple congenital anomalies-hypotonia-seizures syndrome 3	No management implication
KIF1A	Mental retardation, Neuropathy hereditary sensory, Spastic paraplegia	No management implication
SLC6A8	Creatine Deficiency Syndromes	Creatine, L-arginine and L-glycine supplementation[12, 19]
ZEB2	Mowat-Wilson Syndrome	No management implication, Observational clinical trial[13]
PCDH19	Dravet-like Syndrome	Most effective AED clobazam and bromide[14], Interventional clinical trials[30,31]
MEF2C	Mental retardation	No management implication
DCX	Lissencephaly, Subcortical laminal heterotopia	No management implication
PPT1	Neuronal ceroid lipofuscinosis type I	Biochemical testing assessing enzymatic activity[7]
RNASEH2B	Aicardi-Goutières syndrome	No management implication, Possible management with Ruxolitinib[15], Interventional clinical trials[33]
1p36	1p36 deletion syndrome	No management implication, Observational clinical trial[34]
1q25.2-q44; Xq23-q28	Unknown: multiple gene deletion	No management implication

Supplemental table 3 References:

1. ClinicalTrials.gov. National Institute of Health. Available at: https://clinicaltrials.gov/ct2/results?cond=Rett+Syndrome&Search=Apply&age_v=&gndr=&type=Intr&rslt=. Accessed March 13, 2020.
2. U.S. Food and Drug Administration. FDA approves first treatment for a form of Batten disease. 2017. Available at: https://www.fda.gov/newsevents/newsroom/pressannouncements/ucm555613.htm. Accessed March 10, 2020
3. Johnson TB, Cain JT, White KA, Ramirez-Montealegre D, Pearce DA, Weimer JM. Therapeutic landscape for Batten disease: current treatments and future prospects. Nat Rev Neurol 2019 March;15(3): 161–178.
4. ClinicalTrials.gov. National Institute of Health. Available at: https://clinicaltrials.gov/ct2/results?cond=CDKL5&Search=Apply&age_v=&gndr=&type=Intr&rslt=. Accessed: March 13, 2020.
5. EU Clinical Trials Register. Heads of Medicines Agency. Available at: https://www.clinicaltrialsregister.eu/ctr-search/search?query=SCN2A. Accessed: Feb 20, 2020.
6. Gomathi M, Subramanian P, Balachandar V. Drug Studies on Rett Syndrome: From Bench to Bedside. J Autism Dev Disord. 2020 Feb 3 [Epub ahead of print].
7. Mole SE, Williams RE. Neuronal Ceroid-Lipofuscinoses. 2001 Oct 10 [Updated 2013 Aug 1]. In: Adam MP, Ardinger HH, Pagon RA, et al., editors. GeneReviews® [Internet]. Seattle (WA): University of Washington, Seattle; 1993-2020. Available from: https://www.ncbi.nlm.nih.gov/books/NBK1428/. Accessed: March 20, 2020.
8. Miller IO, Sotero de Menezes MA. SCN1A Seizure Disorders. 2007 Nov 29 [Updated 2019 Apr 18]. In: Adam MP, Ardinger HH, Pagon RA, et al., editors. GeneReviews® [Internet]. Seattle (WA): University of Washington, Seattle; 1993-2020. Available from: https://www.ncbi.nlm.nih.gov/books/NBK1318/. Accessed March 10, 2020.
9. ClinicalTrials.gov. National Institute of Health. Available at: https://clinicaltrials.gov/ct2/results?cond=SCN2A&Search=Apply&age_v=&gndr=&type=Obsr&rslt=. Accessed March 13, 2020.
10. Sanders SJ, Campbell AJ, Cottrell JR, Moller RS, Wagner FF, Auldridge AL, et al. Progress in Understanding and Treating SCN2A-Mediated Disorders. Trends Neurosci 2018;41(7): 442–56.

11. Tabarki B, Al-Hashem A, Alfadhel M. Biotin-Thiamine-Responsive Basal Ganglia Disease. 2013 Nov 21. In: Adam MP, Ardinger HH, Pagon RA, et al., editors. GeneReviews® [Internet]. Seattle (WA): University of Washington, Seattle; 1993-2020. Available from: https://www.ncbi.nlm.nih.gov/books/NBK169615/ Accessed: March 10, 2020
12. Dunbar M, Jaggumantri S, Sargent M, Stockler-Ipsiroglu S, van Kamebeek CD. Treatment of X-Linked Creatine Transporter (SLC6A8) Deficiency: Systematic Review of the Literature and Three New Cases. Mol Genet Metab. 2014;112(4): 259–74.
13. World Health Organization. International Clinical Trials Registry Platform. Available at: https://apps.who.int/trialsearch/Trial2.aspx?TrialID=JPRN-UMIN000022565. Accessed: March 13, 2020.
14. Lotte J, Bast T, Borusiak P, Coppola A, Cross JH, Dimova P, et al. Effectiveness of antiepileptic therapy in patients with PCDH19 mutations. Seizure 2016;35:106-10.
15. Tüngler V, Konig N, Engel K, Smitka M, Ungerath K, von der Hagen M, et al. Effects of Janus Kinase Inhibition in Two Children with Aicardi-Goutières Syndrome. Neuropediatrics 2017;48(S 01): S1–45.
16. Khaikin Y, Mercimek-Mahmutoglu S. STXBP1 Encephalopathy with Epilepsy. 2016 Dec 1. In: Adam MP, Ardinger HH, Pagon RA, et al., editors. GeneReviews® [Internet]. Seattle (WA): University of Washington, Seattle; 1993-2020. Available from: https://www.ncbi.nlm.nih.gov/books/NBK396561/ Accessed: March 10, 2020
17. Miller DT, Chung W, Nasir R, et al. 16p11.2 Recurrent Microdeletion. 2009 Sep 22 [Updated 2015 Dec 10]. In: Adam MP, Ardinger HH, Pagon RA, et al., editors. GeneReviews® [Internet]. Seattle (WA): University of Washington, Seattle; 1993-2020. Available from: https://www.ncbi.nlm.nih.gov/books/NBK11167/ Accessed: March 10, 2020
18. Tabarki B, Al-Hashem A, Alfadhel M. Biotin-Thiamine-Responsive Basal Ganglia Disease. 2013 Nov 21. In: Adam MP, Ardinger HH, Pagon RA, et al., editors. GeneReviews® [Internet]. Seattle (WA): University of Washington, Seattle; 1993-2020. Available from: https://www.ncbi.nlm.nih.gov/books/NBK169615/ Accessed: March 10, 2010
19. Mercimek-Mahmutoglu S, Salomons GS. Creatine Deficiency Syndromes. 2009 Jan 15 [Updated 2015 Dec 10]. In: Adam MP, Ardinger HH, Pagon RA, et al., editors. GeneReviews® [Internet]. Seattle (WA): University of Washington, Seattle; 1993-2020. Available from: https://www.ncbi.nlm.nih.gov/books/NBK3794/ Accessed: March 10, 2020
20. ClinicalTrials.gov. National Institute of Health. Available at: https://clinicaltrials.gov/ct2/results?cond=Neuronal+Ceroid+Lipofuscinosis+CLN7&Search=Apply&age_v=&gndr=&type=Obsr&rslt=. Accessed: March 13, 2020.
21. Hehr U, Uyanik G, Aigner L, et al. DCX-Related Disorders. 2007 Oct 19 [Updated 2019 Feb 7]. In: Adam MP, Ardinger HH, Pagon RA, et al., editors. GeneReviews® [Internet]. Seattle (WA): University of Washington, Seattle; 1993-2020. Available from: https://www.ncbi.nlm.nih.gov/books/NBK1185/ Accessed: March 10, 2020
22. ClinicalTrials.gov. National Institute of Health. Available at: https://clinicaltrials.gov/ct2/results?cond=chd2&term=&cntry=&state=&city=&dist=. Accessed: March 13, 2020.
23. ClinicalTrials.gov. National Institute of Health. Available at: https://clinicaltrials.gov/ct2/results?cond=UBE3a&age_v=&gndr=&type=Intr&rslt=&Search=Apply. Accessed March 13, 2020.
24. EU Clinical Trials Register. Heads of Medicines Agency. Available at: https://www.clinicaltrialsregister.eu/ctr-search/search?query=angelman. Accessed: March 13, 2020.
25. ClinicalTrials.gov. National Institute of Health. Available at: https://clinicaltrials.gov/ct2/results?cond=tuberous+sclerosis&age_v=&gndr=&type=Intr&rslt=&Search=Apply. Accessed: March 13, 2020.
26. EU Clinical Trials Register. Heads of Medicines Agency. Available at: https://www.clinicaltrialsregister.eu/ctr-search/search?query=tuberous+sclerosis. Accessed: March 13, 2020.
27. ClinicalTrials.gov. National Institute of Health. Available at: https://clinicaltrials.gov/ct2/results?cond=SYNGAP1&term=&cntry=&state=&city=&dist=. Accessed: March 13, 2020.
28. ClinicalTrials.gov. National Institute of Health. Available at: https://clinicaltrials.gov/ct2/results?cond=16P11.2+Deletion+Syndrome&term=&cntry=&state=&city=&dist=. Accessed March 13, 2020.
29. EU Clinical Trials Register. Heads of Medicines Agency. Available at: https://www.clinicaltrialsregister.eu/ctr-search/search?query=16p11.2. Accessed: March 13, 2020.
30. ClinicalTrials.gov. National Institute of Health. Available at: https://clinicaltrials.gov/ct2/results?cond=PCDH19&age_v=&gndr=&type=Intr&rslt=&Search=Apply. Accessed: March 13, 2020.
31. EU Clinical Trials Register. Heads of Medicines Agency. Available at: https://www.clinicaltrialsregister.eu/ctr-search/search?query=PCDH19. Accessed: March 13, 2020.
32. World Health Organization. International Clinical Trials Registry Platform. Available at: https://apps.who.int/trialsearch/Trial2.aspx?TrialID=JPRN-UMIN000022565. Accessed: March 13, 2020.
33. ClinicalTrials.gov. National Institute of Health. Available at: https://clinicaltrials.gov/ct2/results?cond=Aicardi+Goutieres+Syndrome&age_v=&gndr=&type=Intr&rslt=&Search=Apply. Accessed: March 13, 2020.
34. ClinicalTrials.gov. National Institute of Health. Available at: https://clinicaltrials.gov/ct2/results?cond=1p36&age_v=&gndr=&type=Obsr&rslt=&Search=Apply. Accessed: March 13, 2020.
